# Atomistic modelling and NMR studies reveal that gallium can target the ferric PQS uptake system in *P. aeruginosa* biofilms

**DOI:** 10.1099/mic.0.001422

**Published:** 2023-12-20

**Authors:** Oliver J. Hills, Isaac O.K. Noble, Alex Heyam, Andrew J. Scott, James Smith, Helen F. Chappell

**Affiliations:** ^1^​ School of Food Science and Nutrition, University of Leeds, Woodhouse Lane, Leeds, LS2 9JT, UK; ^2^​ School of Chemistry, University of Leeds, Woodhouse Lane, Leeds, LS2 9JT, UK; ^3^​ School of Chemical and Process Engineering, University of Leeds, Woodhouse Lane, Leeds, LS2 9JT, UK

**Keywords:** PQS, gallium, DFT, NMR

## Abstract

Intravenous gallium nitrate therapy is a novel therapeutic strategy deployed to combat chronic *Pseudomonas aeruginosa* biofilm infections in the lungs of cystic fibrosis (CF) patients by interfering with iron (Fe^3+^) uptake. The therapy is a source of Ga^3+^, which competes with Fe^3+^ for siderophore binding, subsequently disrupting iron metabolism and inhibiting biofilm proliferation *in vivo*. It was recently demonstrated that the *Pseudomonas* quinolone signal (PQS) can chelate Fe^3+^ to assist in bacterial iron uptake. However, it is unknown whether exogenous gallium also targets [Fe(PQS)_3_] uptake, which, in turn, would extend the mechanism of gallium therapy beyond siderophore competition, potentially supporting use of the therapy against *P. aeruginosa* mutants deficient in siderophore uptake proteins. To that end, the thermodynamic feasibility of iron-for-gallium cation exchange into [Fe(PQS)_3_] was evaluated using quantum chemical density functional theory (DFT) modelling and verified experimentally using ^1^H nuclear magnetic resonance (NMR). We demonstrate here that Ga^3+^ can strongly bind to three PQS molecules and, furthermore, displace and substitute Fe^3+^ from the native chelate pocket within PQS complexes, through a Trojan horse mechanism, retaining the key structural features present within the native ferric complex. As such, [Fe(PQS)_3_] complexes, in addition to ferric–siderophore complexes, represent another target for gallium therapy.

## Data Summary

The authors confirm all supporting data, code and protocols have been provided within the article or through electronic Supplementary Data files. The data associated with this paper are openly available from the University of Leeds Data Repository: https://doi.org/10.5518/1447.

## Introduction

Cystic fibrosis (CF) is an autosomal recessive, monogenetic disorder caused by mutations within the cystic fibrosis transmembrane conductance regulator (*CFTR*) gene located on chromosome 7. These mutations prevent Golgi-based translocation of *CFTR* copies (irrespective of their functionality) to cell surface membranes, where they are required for the facilitated diffusion of Cl^−^ out of cells [1]. This causes the destabilization of membrane ion and water potential gradients, leading to dysregulated absorption and release of ions, as well as secretion of glycoproteins, such as mucins, from cells [2].

The most common CF-causing mutation, ΔF508-CFTR, interferes with ferric ion (Fe^3+^) metabolism, leading to elevated concentrations of Fe^3+^ within CF airways [3]. As a result, CF airways are a prime infection site for bacteria that metabolize Fe^3+^ for growth and survival, such as *Pseudomonas aeruginosa* – a Gram-negative, opportunistic pathogen capable of forming persistent biofilms, and one of the most dangerous pathogens associated with CF disease [3, 4].

The development of *P. aeruginosa* biofilms within CF lungs is triggered by quorum sensing autoinducer molecules secreted by the bacteria following attachment. The *Pseudomonas* quinolone signal (PQS) secreted by mucoid *P. aeruginosa* [5, 6] is responsible for regulating mid-to-late stage [7] mucoid *P. aeruginosa* biofilm proliferation and maintenance [7, 8]. PQS has the additional function of acting as an Fe^3+^ chelator – transcriptome analysis of *P. aeruginosa* grown in the presence of exogenous PQS revealed that there was an upregulation of genes related to Fe^3+^ acquisition [9]. This was supported by mass spectrometry analyses and iron chelation assays, indicating that PQS directly chelates Fe^3+^ in a 3 : 1 complex ([Fe(PQS)_3_]) at physiological pH, and behaves as an iron storage vessel [9, 10].

Recently, siderophore-deficient *P. aeruginosa* cells were found to take up Fe^3+^ using PQS as a delivery vessel. Briefly, the H3-T6SS (type VI secretion system) produces a substrate, TseF, capable of binding PQS–Fe^3+^ adducts and facilitating cellular Fe^3+^ uptake by interacting with the siderophore uptake proteins FptA and/or OprF [11]. These findings, and that of Fe^3+^ acting as an environmental signal for the proliferation of a mucoid biofilm [12], suggest that a strategy for reducing the proliferation of chronic *P. aeruginosa* infections in CF airways could involve the sequestration of Fe^3+^ from biofilms [13]. Indeed, removing Fe^3+^ from the extracellular medium has been shown to impede mucoid *P. aeruginosa* biofilm development *in vitro* [14, 15].

In the last decade, there has been increasing interest in gallium as a potential antimicrobial agent [16–18]. For example, gallium–hydroxyapatite nanocomposites were shown to have good antimicrobial effects against *P. aeruginosa* and low cytotoxicity in human lung fibroblasts IMR-90 and mouse fibroblasts L929 during *in vitro* tests [19].

More recently, there have been phase 1 and 2 clinical trials [20, 21] (conducted in CF patients) of intravenous gallium nitrate therapy (IGNT), a promising emerging therapeutic strategy for combatting Fe^3+^ uptake and metabolism *in vivo* [16]. *P. aeruginosa* deploys siderophores with high Fe^3+^-binding affinities to sequester available Fe^3+^ from the surrounding extracellular medium [12] and the high structural similarity between high-spin Fe^3+^ ions (ionic radius: 0.645 Å) and Ga^3+^ ions (ionic radius: 0.620 Å) [22] permits Ga^3+^ from the IGNT to bind siderophores in preference to Fe^3+^ [23]. This is referred to as the so-called Trojan horse mechanism and, ultimately, inhibits *P. aeruginosa* growth [23–25] and biofilm proliferation [26]. Ga^3+^ has also proven capable of forming complexes with synthesized quinolones in a near identical way to Fe^3+^ [27]. Despite these insights, it is currently unknown whether Ga^3+^ can also target Fe^3+^ bound to PQS. If possible, this would support the usage of gallium therapy against *P. aeruginosa* mutants deficient in siderophore uptake protein production.

Here, we utilize density functional theory (DFT)-based geometry optimization and ^1^H nuclear magnetic resonance (NMR) spectroscopy to show that Ga^3+^ is capable of forming stable coordinate complexes with PQS and, critically, displacing Fe^3+^ from [Fe(PQS)_3_].

## Methods

### Computational

The thermodynamic feasibility of the formation of [Fe(PQS)_3_] was calculated using quantum chemical DFT modelling. The thermodynamic stability of initial [Fe(PQS)_3_] was calculated through evaluating a formation energy according to Equation 1:


(1) $$E_{f}=E_{\left\{iron-PQS\;complex\right\}}-\left(3E_{PQS}+\mu_{Fe}\right)$$

Within Equation 1, *E*_{iron–PQS complex}_ represents the energy of the [Fe(PQS)_3_] system, *E*_PQS_ represents the energy of the PQS molecule that has been modified to remove the hydrogen atom from the 3′ OH functional group (see Results and Discussion for further discussion), and *μ*_Fe_ represents the chemical potential of iron. A negative *E*_*f*_ value would confirm that a [Fe(PQS)_3_] system is thermodynamically stable.

The thermodynamic feasibility of the iron-for-gallium cation exchange into [Fe(PQS)_3_] was computed in a similar manner. The thermodynamic stability of the product complex, [Ga(PQS)_3_], relative to [Fe(PQS)_3_], was calculated according to Equation 2:


(2) $$E_{f}=E_{\left\{gallium-PQS\;complex\right\}}-\left(E_{\left\{iron-PQS\;complex\right\}}+\mu_{Ga}-\mu_{Fe}\right)$$

Within Equation 2, *E*_{gallium–PQS complex}_ represents the energy of [Ga(PQS)_3_], *E*_{_ *_iron–PQS complex_*_}_ represents the energy of initial [Fe(PQS)_3_], *μ*_Ga_ represents the chemical potential of gallium and *μ*_Fe_ the chemical potential of iron. A negative *E*_*f*_ value would suggest that the iron-for-gallium cation exchange into [Fe(PQS)_3_] complexes is thermodynamically feasible, with the potential to occur *in vitro* and *in vivo*.

All energies and chemical potentials were calculated using the plane wave, pseudopotential DFT method, as implemented in the CASTEP code [28]. A convergence tested cut-off energy of 950 eV was used alongside a Monkhorst–Pack k-point grid of 1×1×1 to sample the Brillouin zone [29]. On-the-fly ultrasoft pseudopotentials [30] and the PBE-TS exchange-correlation functional were employed [31, 32]. The SCF tolerance for the electronic minimizations was set to 1×10^−7^ eV atom^−1^ and the energy, force and displacement tolerances for the geometry optimizations were set to 1×10^−5^ eV atom^−1^, 0.03 eV Å^−1^ and 1×10^−3^ Å, respectively. Following each geometry optimization, Mulliken bond populations [33] were calculated to classify the nature of bonding in each of the complexed structures. All atomistic models were created and visualized using CrystalMaker [34]. The chemical potentials for gallium and iron were calculated from their respective 0 K energy per atom of the pure metals in their lowest energy configurations, namely orthorhombic (alpha) gallium and (ferromagnetic) cubic (BCC, alpha) iron.

### Experimental

2-heptyl-3-hydroxy-4(1 h)-quinolone (PQS) was purchased from Sigma-Aldrich (product no. 94 398, purity 96.0+%). Gallium trichloride (GaCl_3_) was purchased from Thermo Fisher Scientific (catalogue no. 444 100 050, purity 99.99+%). Ferric chloride hexahydrate (FeCl_3_·6H_2_O) was purchased from Fluorochem Drug Discovery (product code F094731, purity 99.0 %).

Five millimolar stock solutions of PQS, FeCl_3_ and GaCl_3_ were made by dissolving 0.01 g of each reagent in the required volume of deuterated methanol (Eurisotop). NMR samples for [Fe(PQS)_3_] and [Ga(PQS)_3_] were prepared by mixing the stock solutions with deuterated methanol in Wilmad 528-PP NMR tubes.

^1^H NMR spectra were recorded on a Bruker 500 MHz Avance Neo spectrometer equipped with a triple-resonance room temperature probe at a temperature of 298 K. One hundred and twenty-eight scans were recorded using Bruker pulse sequence zg30 with an acquisition time of 3.2 s and a relaxation delay of 1 s. Data were then processed using Bruker Topspin software using line broadening of 0.3 Hz and were referenced relative to the methyl signal of deuterated methanol.

## Results and discussion

### Iron–PQS system

At physiological pH, it has been demonstrated that three PQS molecules can effectively chelate a single Fe^3+^ centre, facilitated by the PQS 4′ carbonyl and 3′ hydroxyl functional groups [10]. By contrast, HHQ, a structural analogue of PQS that lacks a 3′ OH, cannot bind Fe^3+^, drawing attention to the critical role of the 3′ hydroxyl in iron sequestration [10]. Furthermore, DFT and infrared spectroscopy have shown that Fe^3+^ and Ga^3+^ form complexes with synthetic quinolones in a 1 : 3 ratio, with each ligand binding in a bidentate fashion [27]. However, given that the phenolate group offers a key coordination mode in pyochelin [35] as well as in ferric hydroxyquinolone [36], it is likely that an anionic phenolate group, rather than the (acidic) hydroxyl group, is involved in the [Fe(PQS)_3_] complexation event, giving an overall charge neutral complex. Considering these findings, we constructed a model of [Fe(PQS)_3_] in which the 3′ OH groups of the PQS molecules were dehydrogenated, permitting three PQS molecules to complex with a single Fe^3+^ as bidentate ligands. Furthermore, PQS bidentate ligands can chelate Fe^3+^ in either an octahedral low-spin (S=1/2, singlet) or octahedral high-spin (S=5/2, sextet) geometry. Initial DFT optimizations using CASTEP showed the high-spin [Fe(PQS)_3_] complex to be 0.057 eV (5.50 KJ mol^−1^), more stable than the low-spin complex (data not shown). As such, it was considered the dominant spin polarization.

**Fig. 1.**
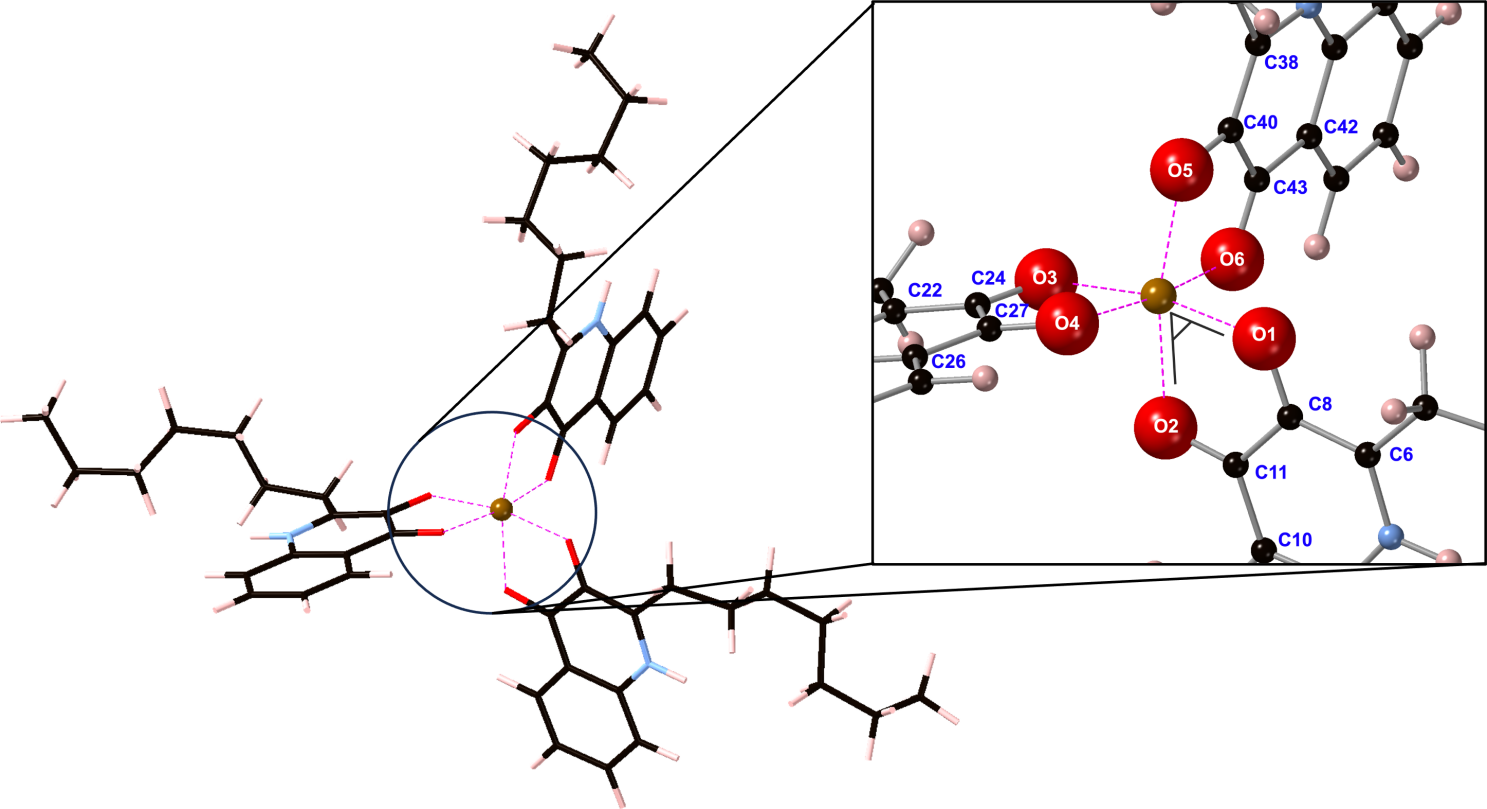
Geometry-optimized [Fe(PQS)_3_]. The geometry-optimized, energetically favourable output for [Fe(PQS)_3_] is displayed on the left. An expanded view of the Fe^3+^ coordination sphere is displayed on the right with the phenolate O–Fe^3+^–O ketone bite angle marked in black. Carbon atoms are shown in black, oxygen in red, hydrogen in pink and Fe^3+^ in brown. Ionic interactions between PQS and the Fe^3+^ centre are illustrated by dashed pink lines.

Geometry optimization of the [Fe(PQS)_3_] model resulted in an energetically favourable formation energy (*E*_*f*_ *= –*4.39 eV), providing confirmation of the stability of the initial complex (Fig. 1). The complex exhibited a pseudo-octahedral geometry with O–Fe^3+^–O bond angles averaging at 90.18 (7.01)°, (Table S1, available in the online version of this article). This was corroborated by examining the Fe^3+^–O–C–C dihedral angles (Table 1). The average phenolate O–Fe^3+^–O ketone bite angle (of each ligand) was 79.88°.

**Table 1.** Fe^3+^–O–C–C and Ga^3+^–O–C–C dihedral angles (°) present within [Fe(PQS)_3_] and [Ga(PQS)_3_] following iron-for-gallium substitution into the [Fe(PQS)_3_]. Numbering of atoms given in Figs. 1 and 2

Plane	Dihedral angle (°)	Plane	Dihedral angle (°)
Fe–O1–C8–C6	+172.45	Ga–O1–C8–C6	+172.90
Fe–O2–C11–C10	−172.12	Ga–O2–C11–C10	−172.54
Fe–O3–C24–C22	−171.41	Ga–O3–C24–C22	−172.25
Fe–O4–C27–C26	+172.25	Ga–O4–C27–C26	+173.50
Fe–O5–C40–C38	−177.10	Ga–O5–C40–C38	−176.42
Fe–O6–C43–C42	+179.35	Ga–O6–C43–C42	+179.40

**Fig. 2.**
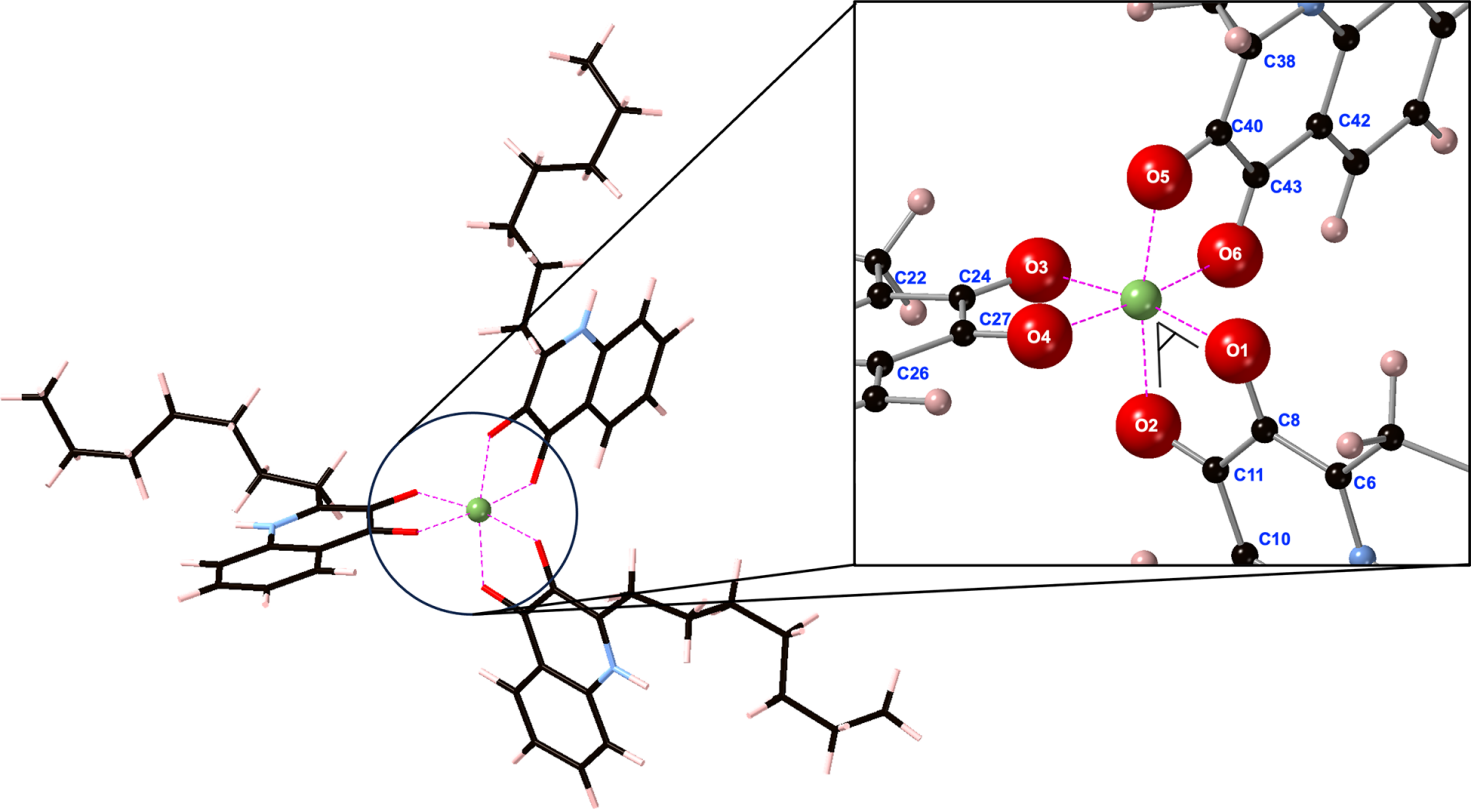
[Ga(PQS)_3_] following the iron-for-gallium cation exchange into high-spin [Fe(PQS)_3_]. An expanded view of the Ga^3+^ coordination sphere is displayed on the right. The phenolate O–Ga^3+^–O ketone bite angle is marked in black. Carbon atoms are shown in black, oxygen in red, hydrogen in pink and gallium in green. Ionic interactions between PQS and the Ga^3+^ centre are illustrated by dashed pink lines.

### Gallium-for-Iron PQS system

The structure of [Ga(PQS)_3_] following the iron-for-gallium cation exchange into high-spin [Fe(PQS)_3_] is given in Fig. 2.

Within [Ga(PQS)_3_] (Fig. 2), PQS 3′ phenolate groups established more stable interactions with the central Ga^3+^ (average length and population equal to 1.99 Å and 0.32 |e|, respectively) relative to the 4′ ketone groups (average length and population equal to 2.05 Å and 0.27 |e|, respectively). The phenolate O–Ga^3+^ interactions were on average 0.06 Å shorter than the ketone O–Ga^3+^ contacts, which matched the lengths observed in other gallium–organic ligand systems, namely the crystal structures of gallium–chrysin complexes and DFT optimized gallium–5-hydroxyflavone complexes [37, 38]. The average phenolate O–Ga^3+^–O ketone bite angle (Fig. 2) within [Ga(PQS)_3_] was 82.87°, also agreeing well with the equivalent bite angles observed within gallium–chrysin complexes and DFT-optimized gallium–5-hydroxyflavone complexes [37, 38].

The average of all the O–Ga^3+^–O bond angles, including the angles formed between neighbouring PQS ligands, was 90.19 (5.28)° (Table S1), indicating that the geometry adopted by the central gallium ion slightly deviated from pure octahedral coordination. This was further corroborated by analysing the Ga^3+^–O–C–C dihedral angles, given in Table 1.

Ga^3+^–O–C–C dihedral angles equal to ± 180° would indicate that the donating oxygen atoms from a single bidentate PQS ligand lie within the same plane. As shown in Table 1, these angles deviated by up to 7.75° from 180°, further highlighting that both [Ga(PQS)_3_] and [Fe(PQS)_3_] produce distorted octahedra with the PQS ligands adopting a quasi-planar coordination geometry.

Encouragingly, this substitution was thermodynamically stable, with Equation 2 returning a negative formation energy of −1.92 eV. As such, exogenous Ga^3+^ can favourably substitute Fe^3+^ from its native binding site within PQS complexes. Once substituted, Ga^3+^ forms a very stable complex with PQS. Using Equation 1, the formation energy of a native [Ga(PQS)_3_] system, formed without substituting for iron, was −6.31 eV; more stable than [Fe(PQS)_3_] (−4.39 eV). The global (all atom) RMSD, following alignment of the high-spin [Fe(PQS)_3_] and [Ga(PQS)_3_] complexes, was equal to 0.062 Å, showing a very high level of structural similarity between the initial high-spin [Fe(PQS)_3_] and [Ga(PQS)_3_] complexes following the cation exchange, offering a prime demonstrator of the Trojan horse mechanism exhibited by Ga^3+^. In other words, gallium ions were able to establish nearly identical complex geometries within ferric uptake systems.

### PQS molecules chelate Ga^3+^ ions *in vitro*

**Fig. 3.**
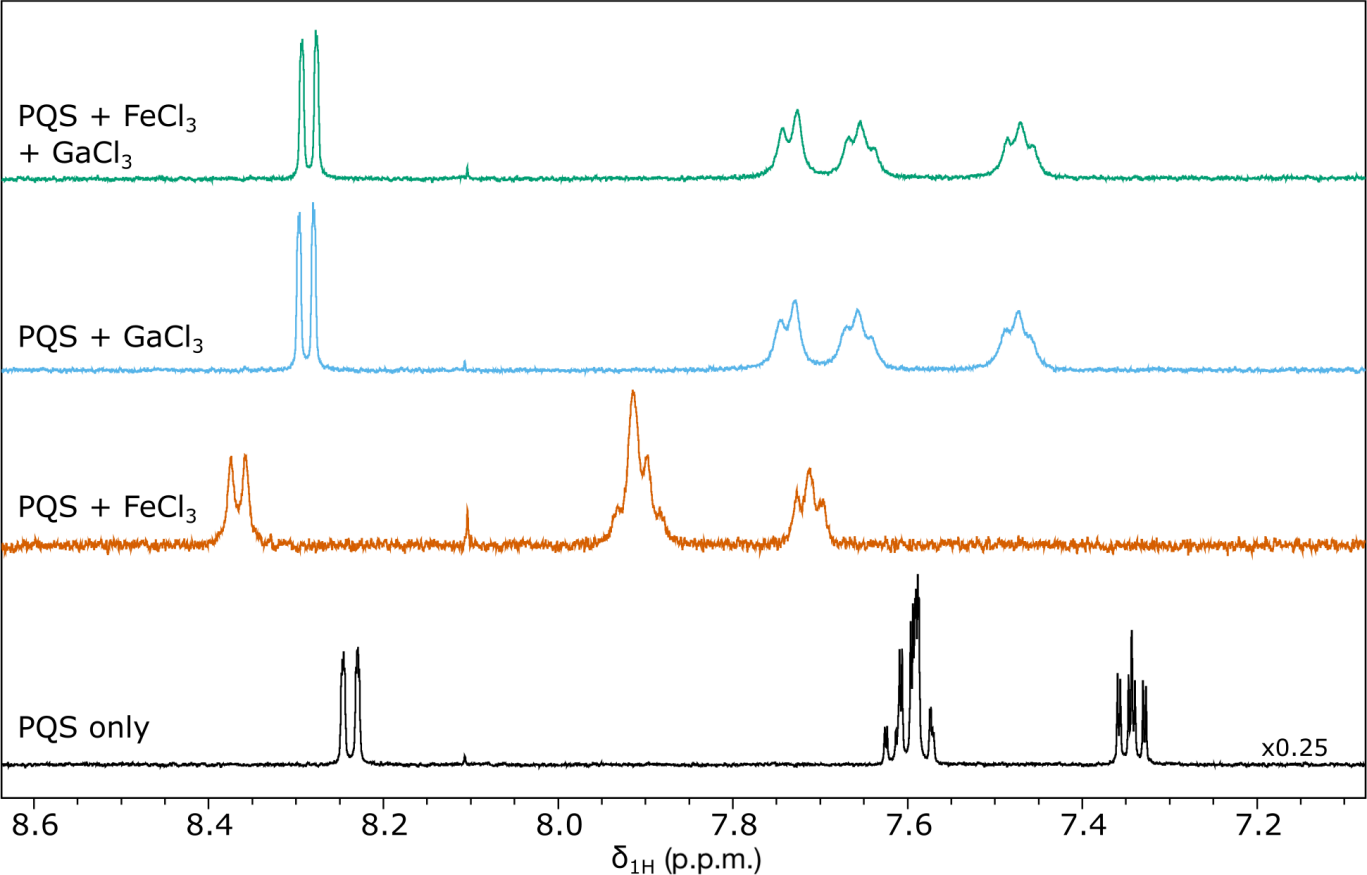
PQS molecules chelate Ga^3+^ ions. ^1^H NMR spectra for 1 mM PQS either alone (black) or mixed with either 1 mM FeCl3 (orange), 1 mM GaCl_3_ (blue), or 1 mM FeCl_3_ and 1 mM GaCl_3_ (green).

To validate the formation of [Ga(PQS)_3_] and the substitution proposed above *in vitro*, ^1^H NMR spectroscopy was conducted on samples containing 1 mM PQS, either alone or mixed with 1 mM FeCl_3_ or 1 mM GaCl_3_. Three or four resonance signals were observed in the aromatic region, corresponding to the four aromatic protons of PQS (Fig. 3, Table S2). Only three resonance signals were visible in the PQS-only and PQS+FeCl_3_ spectra due to strong coupling between two resonance signals with similar chemical shift – resonance signal integration and 2D (^1^H,13C)-HSQC spectroscopy showed that all four protons were present (Table S2, Fig. S1a).

Relative to PQS alone, all signals broadened and increased in chemical shift when FeCl_3_ was added, indicating the formation of the previously reported [Fe(PQS)_3_] (Fig. 3) [10]. A similar shift and broadening was observed when GaCl_3_ was added to PQS, demonstrating that PQS molecules can also chelate gallium ions to form [Ga(PQS)_3_], as predicted by the modelling work above (*E*_*f*_=−6.31 eV).

Equation 2 predicted that it would be energetically favourable for [Ga(PQS)_3_] to form via an iron-for-gallium transition substitution mechanism. To test this *in vitro*, GaCl_3_ was added to a mixed solution containing PQS and FeCl_3_ to give a final concentration of 1 mM each. The subsequent ^1^H NMR spectrum of this solution matched that of [Ga(PQS)_3_] and not that of [Fe(PQS)_3_] (Figs 3 and S1b), confirming our hypothesis. This was also consistent with the colour of the solution: the [Fe(PQS)_3_] solution had a reddish-pink colour (as previously reported [9, 39]) which faded when GaCl_3_ was added (data not shown).

### Conclusion

Given the near identical geometries of high-spin [Fe(PQS)_3_] and [Ga(PQS)_3_], it is entirely conceivable that TseF–PQS–Ga^3+^ adducts could trigger gallium uptake in a similar fashion to iron uptake [11]. Our theoretical simulations show the thermodynamic feasibility of this iron-for-gallium substitution into ferric PQS complexes, suggesting a previously unknown mechanism of action that extends past siderophore competition. We have confirmed these theoretical predictions using ^1^H NMR, clearly showing that not only do Ga–PQS complexes form, but that gallium readily displaces iron from pre-formed Fe–PQS systems. Understanding the chemistry that influences the efficiency of this substitution can inform the (re)design of gallium therapy.

## Supplementary Data

Supplementary material 1
