# Supplementary material for: Atomistic modelling and NMR studies reveal that gallium can target the ferric PQS uptake system in P. aeruginosa biofilms
Source: Microbiology (Reading). 2023 Dec 20;169(12):001422. doi: 10.1099/mic.0.001422 (PMC10765035; doi:10.1099/mic.0.001422)

SUPPLEMENTARY INFORMATION

Supplementary Table 1: O-Fe^{3+}-O and O-Ga^{3+}-O bond angles, showing that both ions induce a distorted octahedral geometry. Those marked with an asterisk (*) denote the ion-ligand bite angles.

**Atoms**	**Angle (°)**	**Atoms**	**Angle (°)**
01-Fe-O2*	79.380	01-Ga-O2*	82.191
01-Fe-04	96.094	01-Ga-04	93.387
01-Fe-05	93.203	01-Ga-05	91.453
01-Fe-06	93.689	01-Ga-06	93.969
02-Fe-03	89.564	02-Ga-03	89.017
02-Fe-04	94.223	02-Ga-04	93.542
02-Fe-06	87.986	02-Ga-06	88.071
03-Fe-04*	80.062	03-Ga-04*	83.018
03-Fe-05	98.562	03-Ga-05	98.562
03-Fe-06	90.428	03-Ga-06	89.812
04-Fe-05	98.794	04-Ga-05	95.792
05-Fe-06*	80.196	05-Ga-06*	83.409

Supplementary Table 2: ^{1}H chemical shifts observed for PQS complexes observed by NMR. With the data available, it was not possible to assign the signals to specific atoms in PQS. However, the number of signals and their chemical shifts and integrals is consistent with the 4 aromatic protons of PQS.

	^{1}H chemical shift /ppm (relative peak integral)			
	A	B	C	D
PQS only	8.24 (1)	7.60 (2.10)		7.34 (1.04)
PQS + $FeCl_3$	8.37 (1)	7.91 (2.11)		7.71 (1.08)
PQS + $GaCl_3$	8.29 (1)	7.74 (1.01)	7.66 (1.02)	7.47 (1.00)

Supplementary Figure 1: a) (^{1}H,^{13}C)-HSQC of 1 mM PQS in deuterated methanol, recorded at 298 K on a Bruker 500 MHz Avance Neo NMR spectrometer equipped with DCH cryoprobe. The overlapped ^{1}H signal at 7.6 ppm is now resolved into two separate signals with (^{1}H, ^{13}C) chemical shifts of (7.60, 130.3) ppm and (7.59, 117.4) ppm. b) Complete overlay of ^{1}H NMR spectra of 1 mM PQS with 1 mM $GaCl_3$ (red) and 1 mM PQS with 1 mM $FeCl_3$ and 1 mM $GaCl_3$ (blue). They are identical (apart from minor differences around the residual methanol signals), demonstrating that all PQS is complexed with Ga^{3+} and not Fe^{3+}.

a)

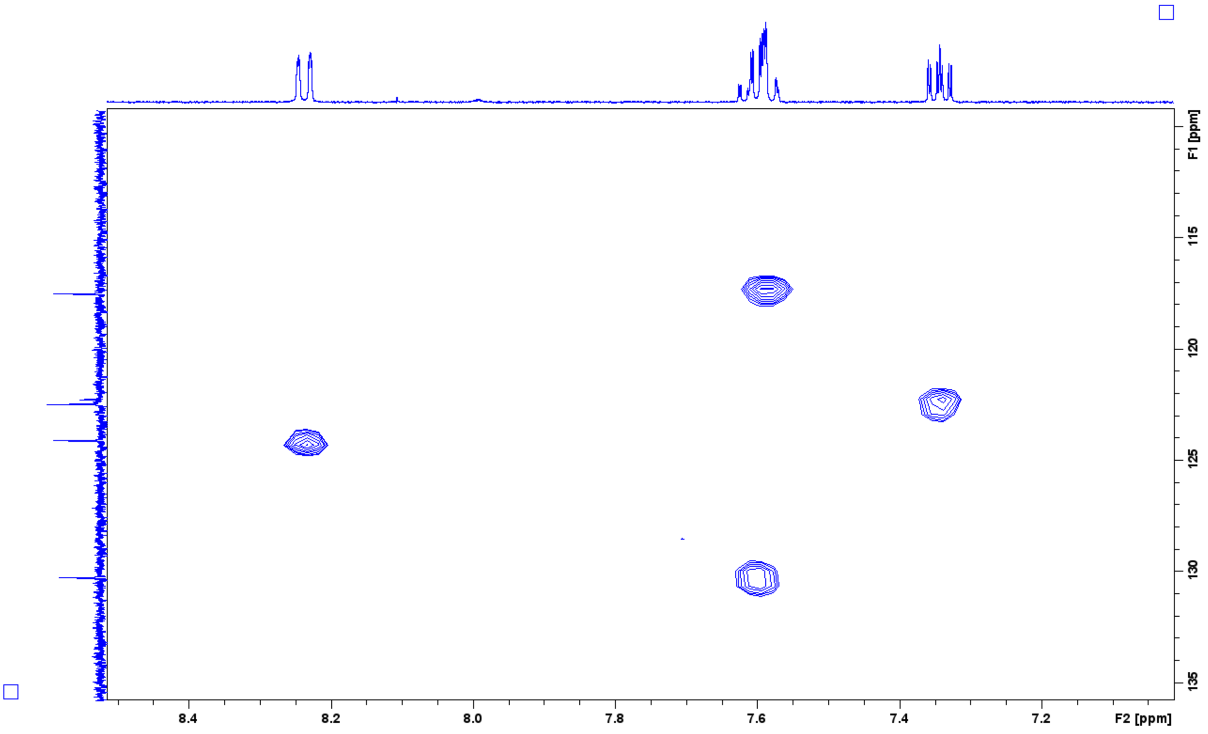

b)

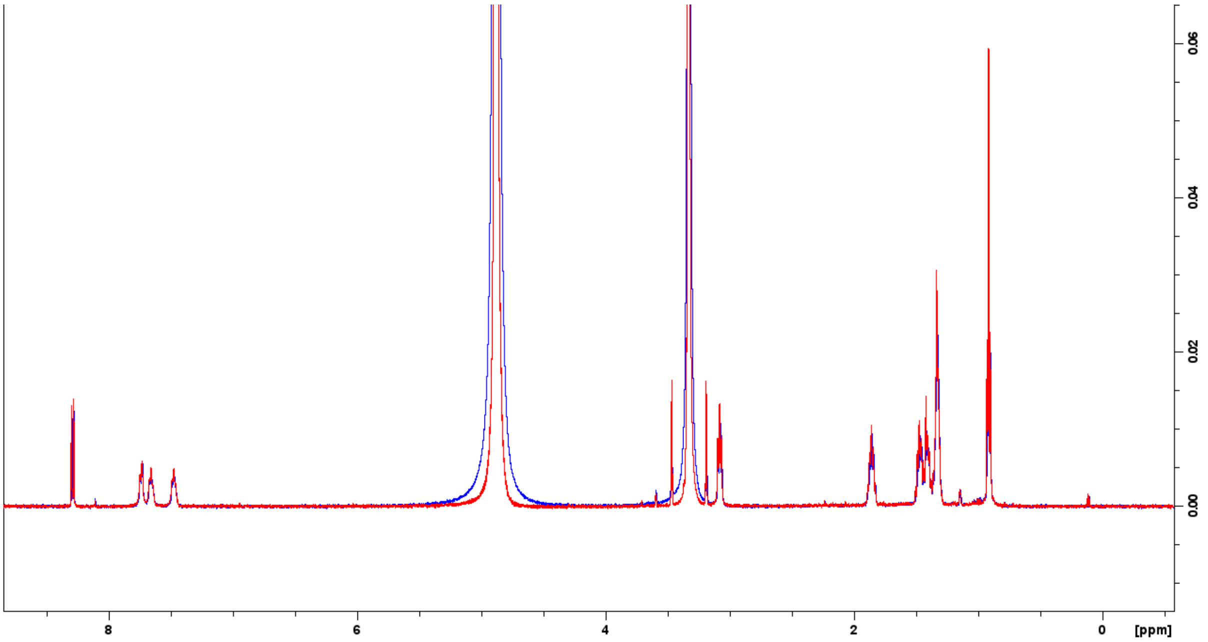

Supplement: Supplementary material 1 [file mic-169-1422-s001.pdf]
